# Detection of influenza A(H3N2) viruses exhibiting reduced susceptibility to the novel cap-dependent endonuclease inhibitor baloxavir in Japan, December 2018

**DOI:** 10.2807/1560-7917.ES.2019.24.3.1800698

**Published:** 2019-01-17

**Authors:** Emi Takashita, Chiharu Kawakami, Hiroko Morita, Rie Ogawa, Seiichiro Fujisaki, Masayuki Shirakura, Hideka Miura, Kazuya Nakamura, Noriko Kishida, Tomoko Kuwahara, Keiko Mitamura, Takashi Abe, Masataka Ichikawa, Masahiko Yamazaki, Shinji Watanabe, Takato Odagiri, on behalf of the Influenza Virus Surveillance Group of Japan

**Affiliations:** 1 Influenza Virus Research Center, National Institute of Infectious Diseases, Tokyo, Japan; 2 Yokohama City Institute of Public Health, Kanagawa, Japan; 3 Eiju General Hospital, Tokyo, Japan; 4 Abe Children’s Clinic, Kanagawa, Japan; 5 Ichikawa Children’s Clinic, Kanagawa, Japan; 6 Zama Children’s Clinic, Kanagawa, Japan; 7 The members of the group are listed at the end of the article

**Keywords:** Influenza virus, cap-dependent endonuclease inhibitor, baloxavir marboxil, baloxavir acid, resistant

## Abstract

The novel cap-dependent endonuclease inhibitor baloxavir marboxil was approved for the treatment of influenza virus infection in Japan in February 2018. Two influenza A(H3N2) viruses carrying an I38T substitution in the polymerase acidic subunit (PA) were detected in baloxavir-treated children in December 2018. This mutation is known to confer reduced susceptibility to baloxavir, and the two mutant viruses exhibited 76- and 120-fold reduced susceptibility to baloxavir.

The novel antiviral drug baloxavir marboxil was approved in Japan on 23 February 2018 for the treatment of influenza virus infection, in patients 12 years and older and children younger than 12 years weighing 10 kg or more; it became available on 14 March 2018 in Japan (Figure). The hydrolysed active form of baloxavir marboxil (baloxavir acid) inhibits the cap-dependent endonuclease of influenza A and B viruses [1]. In Phase II and III clinical trials, I38T, I38F and I38M substitutions in the polymerase acidic subunit (PA) were detected in A(H1N1)pdm09 and A(H3N2) influenza viruses [2,3]. Patients infected with these mutant viruses exhibited prolonged virus shedding, and the median time to symptom alleviation was longer in baloxavir recipients infected with viruses bearing these substitutions than in those infected with viruses that lacked these substitutions [2,3]. Therefore, we conducted nationwide monitoring of the baloxavir susceptibility of circulating influenza viruses by using a combination of phenotypic methods to analyse antiviral susceptibility and genotypic methods to detect amino acid substitutions [4].

**Figure:**
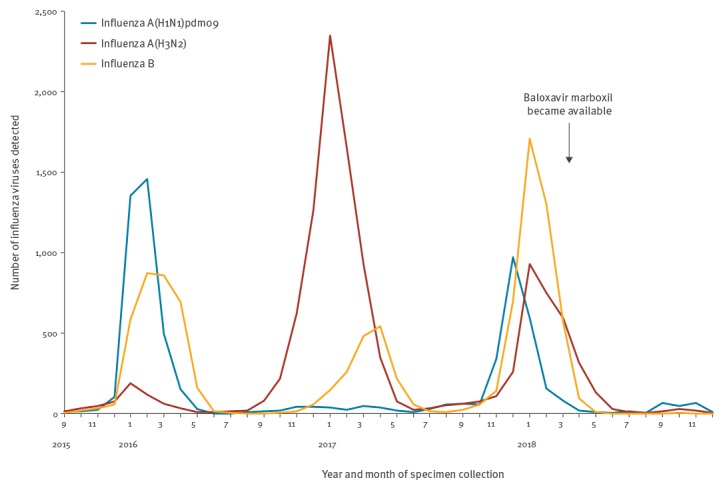
Detection of influenza viruses, Japan, September 2015–December 2018 (n = 28,093)

Monthly reports of influenza virus isolation/detection by the National Epidemiological Surveillance of Infectious Diseases. Baloxavir marboxil became available on 14 March 2018 in Japan.

## Detection of PA I38T mutant influenza A(H3N2) viruses

In December 2018, influenza outbreaks occurred in two primary schools in Yokohama, Japan. We isolated four influenza A(H3N2) viruses, A/YOKOHAMA/133/2018, A/YOKOHAMA/134/2018, A/YOKOHAMA/135/2018 and A/YOKOHAMA/136/2018, from four children during these outbreaks (Table 1). Two patients aged 6 and 7 years, infected with A/YOKOHAMA/133/2018 respectively A/YOKOHAMA/135/2018, were treated with a single oral dose of baloxavir marboxil. The third patient, 7-years-old and infected with A/YOKOHAMA/136/2018, was treated with the neuraminidase (NA) inhibitor oseltamivir, whereas the last patient, aged 7 years and infected with A/YOKOHAMA/134/2018, had no exposure to antiviral drugs before specimen collection. Fever in the two children that received baloxavir resolved within 2 days of baloxavir administration, and in the child that received oseltamivir, it resolved within one day of oseltamivir administration. The child without treatment had a fever at the time of specimen collection.

**Table 1:** Influenza A(H3N2) viruses detected in outbreaks, Yokohama, Japan, December 2018 (n = 4)

GISAID isolate ID	Isolate name	Onset of symptoms	Antiviral treatment	Specimen collection	PA substitution^a^
EPI_ISL_332908	A/YOKOHAMA/133/2018	2 Dec 2018	3 Dec 2018 Baloxavir	6 Dec 2018	I38T (277Y)
EPI_ISL_332910	A/YOKOHAMA/135/2018	4 Dec 2018	4 Dec 2018 Baloxavir	7 Dec 2018	I38T (Y277F)
EPI_ISL_332911	A/YOKOHAMA/136/2018	4 Dec 2018	5–9 Dec 2018 Oseltamivir	7 Dec 2018	38I (277Y)
EPI_ISL_332909	A/YOKOHAMA/134/2018	6 Dec2018	None	7 Dec 2018	38I (Y277F)

GISAID: Global Initiative on Sharing All Influenza Data; PA: polymerase acidic subunit.

^a^The amino acid residue at position 277 is not involved in PA–inhibitor interactions.

Clinical specimens were collected 3 days after baloxavir administration, on day 2 of oseltamivir administration or on the day after onset. Sequencing analysis detected the PA I38T substitution in A/YOKOHAMA/133/2018 and A/YOKOHAMA/135/2018, but not in A/YOKOHAMA/134/2018 or A/YOKOHAMA/136/2018. No amino acid substitutions associated with reduced susceptibility to NA inhibitors were detected. These results demonstrate that PA I38T mutant viruses were isolated from children 3 days after baloxavir administration.

## Antiviral susceptibilities of the PA I38T mutant viruses

We compared the susceptibilities of the PA I38T mutant viruses and the wild-type virus to baloxavir and four NA inhibitors approved in Japan: oseltamivir, peramivir, zanamivir and laninamivir (Table 2). Antiviral susceptibilities were determined by using a focus reduction assay and a fluorescent NA inhibition assay with the NA-Fluor Influenza Neuraminidase Assay Kit (Applied Biosystems, California, United States) as previously described [4]. Baloxavir acid was purchased from MedChemexpress (New Jersey, United States). Oseltamivir carboxylate, peramivir and zanamivir were purchased from Sequoia Research Products (Pangbourne, United Kingdom), and laninamivir was provided by Daiichi Sankyo Co., Ltd. (Tokyo, Japan). Results are expressed as the 50% inhibitory concentration (IC_50_).

**Table 2:** Susceptibility of influenza A(H3N2) viruses detected in outbreaks, Yokohama, Japan, December 2018 (n = 4)

Isolate name	PA substitution	IC_50_, nM				
		Baloxavir	Neuraminidase inhibitors (WHO criteria)			
			Oseltamivir^a^	Peramivir^a^	Zanamivir^a^	Laninamivir^a^
A/YOKOHAMA/133/2018	I38T	227.08	0.41 (NI)	0.16 (NI)	0.88 (NI)	0.76 (NI)
A/YOKOHAMA/135/2018	I38T	144.02	0.25 (NI)	0.15 (NI)	0.81 (NI)	0.92 (NI)
A/YOKOHAMA/136/2018	38I	2.78	0.38 (NI)	0.12 (NI)	0.77 (NI)	1.05 (NI)
A/YOKOHAMA/134/2018	38I	1.02	0.28 (NI)	0.12 (NI)	0.76 (NI)	0.88 (NI)

PA: polymerase acidic subunit; IC_50_: 50% inhibitory concentration; NI: Normal inhibition; WHO: World Health Organization.

^a^The median IC_50_ values of 30 influenza A(H3N2) viruses isolated in the 2018/19 influenza season in Japan to oseltamivir, peramivir, zanamivir and laninamivir were 0.22 ± 0.08, 0.11 ± 0.02, 0.50 ± 0.26 and 0.98 ± 0.26, respectively.

The IC_50_ values of the viruses to baloxavir and the NA inhibitors are shown in Table 2. Both the PA I38T mutant viruses and the wild-type viruses showed normal inhibition with all four NA inhibitors, whereas the PA I38T mutant viruses exhibited 76- and 120-fold higher IC_50_ values to baloxavir compared with the mean value of wild-type viruses. These results indicate that the PA I38T mutant viruses had reduced susceptibility to baloxavir, but remained susceptible to NA inhibitors.

## Discussion

A Phase II clinical trial of baloxavir marboxil was conducted in Japan during the 2015/16 influenza season and Phase III trials were conducted in Japan and the United States in the 2016/17 season [2,3]. The drug was approved in February 2018 in Japan and in October 2018 in the United States. During the 2015/16 and 2016/17 seasons, influenza A(H1N1)pdm09 and A(H3N2) viruses, respectively, predominated in Japan. During the Phase II trial, the PA I38T and I38F substitutions emerged after baloxavir treatment in four (3.6%) of 112 A(H1N1)pdm09 viruses isolated from adults aged 20–64 years [4]. In the Phase III trials, the PA I38T and I38M substitutions emerged in 36 (9.7%) of 370 A(H3N2) viruses obtained from patients aged 12–64 years and in 18 (23.4%) of 77 A(H3N2) viruses obtained from children aged 6 months to < 12 years [2,3]. Cumulative data from clinical trials of oseltamivir, involving almost 2,000 oseltamivir-treated patients, indicate that the incidence of reduced susceptibility to oseltamivir is 0.32% in adults and 4.1% in children (if low-level mutants detected by genotyping alone in mixed virus populations are included, then the corresponding values are 0.4% and 5.4%, respectively) [5]. These results suggest that the incidence of influenza viruses exhibiting reduced susceptibility to baloxavir is higher than that to oseltamivir.

In Japan, baloxavir marboxil became available at the end of the 2017/18 influenza season. We isolated two PA I38T mutant influenza A(H3N2) viruses from baloxavir-treated children in December 2018. PA I38 is highly conserved in influenza A and B viruses [3], and the I38T substitution was not detected among 17,227 PA sequences from A(H3N2) viruses in the National Institute of Allergy and Infectious Diseases (NIAID) Influenza Research Database (IRD) [6]. Furthermore, our sequencing analysis revealed that these two PA I38T mutant viruses possessed different PA sequences and thus originated from different viruses, suggesting no human-to-human transmission. Our findings indicate that these viruses emerged under the selective pressure of baloxavir marboxil. In contrast, no viruses exhibiting reduced susceptibility to NA inhibitors were detected among 90 influenza A viruses tested between September and December 2018 in Japan [7]. These observations suggest that the emergence of PA I38T mutant viruses may increase as the use of baloxavir marboxil increases in the 2018/19 influenza season. Therefore, the baloxavir susceptibility of influenza viruses should be closely monitored.

In vitro studies using the plaque reduction assay revealed that influenza A/WSN/33(H1N1) viruses with the PA I38T or I38F substitutions show 27.2- and 10.6-fold higher EC_50_ values (the 50% effective concentration) to baloxavir compared with the wild-type virus [3]. Furthermore, influenza A/Victoria/3/75(H3N2) viruses with the PA I38T or I38M substitutions showed 56.6- and 13.8-fold higher EC_50_ values, respectively [3]. These results suggest that the PA I38T substitution has a marked impact on baloxavir susceptibility. In the present study, we obtained two influenza A(H3N2) clinical isolates possessing the PA I38T substitution. These viruses showed 76- and 120-fold higher IC_50_ values to baloxavir compared with the mean value of wild-type viruses. Our data thus demonstrate that the PA I38T substitution is associated with reduced susceptibility to baloxavir in currently circulating influenza A(H3N2) viruses.

The Technical Expert Working Group of the World Health Organization’s (WHO) Global Influenza Surveillance and Response System (GISRS) for Surveillance on Antiviral Susceptibility (WHO-AVWG) has established a set of criteria to define the NA inhibitor susceptibility of influenza viruses based on the fold-change in IC_50_ value compared with the median value for viruses from the same type/subtype/lineage [8]. For influenza A virus, use of the terms normal (< 10-fold increase), reduced (10–100-fold increase) and highly reduced (> 100-fold increase) inhibition is recommended when reporting and analysing surveillance data; for influenza B, the same definitions are used but for < 5-fold, 5–50-fold and > 50-fold increases. The WHO-AVWG is currently collecting more data on baloxavir marboxil to establish a similar set of criteria to define baloxavir susceptibility, which should be available in the near future.

In summary, our results indicate that continuous monitoring of the emergence of baloxavir-resistant viruses is important for public health planning and clinical recommendations for antiviral drug use.
